# Safety, Complications, and Procedural Efficiency of Neonatal Circumcision Devices: A Network Meta-Analysis

**DOI:** 10.1590/S1677-5538.IBJU.2026.0167

**Published:** 2026-05-02

**Authors:** Amani N. Alansari, Mohammed A. Mahmoud, Habib Ullah Joya, Hanan Youssif, Mousa M. Ahmeda, Marwa Messaoud

**Affiliations:** 1 Hamad Medical Corporation Department of Pediatric Surgery Doha Qatar Department of Pediatric Surgery, Hamad Medical Corporation, Doha, Qatar; 2 Al-Azhar University Faculty of Medicine Gaza Palestine Faculty of Medicine, Al-Azhar University, Gaza, Palestine; 3 Benghazi Medical Center Department of Pediatric Surgery Benghazi Libya Department of Pediatric Surgery, Benghazi Medical Center, Benghazi, Libya; 4 Fattouma Bourguiba University Hospital Department of Pediatric Surgery Monastir Tunisia Department of Pediatric Surgery, Fattouma Bourguiba University Hospital, Monastir, Tunisia; 5 University of Monastir Faculty of Medicine of Monastir Monastir Tunisia Faculty of Medicine of Monastir, University of Monastir, Monastir, Tunisia

**Keywords:** Foreskin, Network Meta-Analysis [Publication Type], complications [Subheading]

## Abstract

**Purpose:**

This study aims to compare safety, complications, and procedural characteristics of commonly used circumcision devices in neonates and early infants.

**Methods:**

We searched PubMed, EMBASE, Web of Science, and SCOPUS through January 2026 for randomized controlled trials comparing infant circumcision devices (Plastibell™, Mogen™, ShangRing™, AccuCirc™) in infants ≤3 months. The primary outcome was total adverse events; secondary outcomes included bleeding, infection, redundant skin, adhesions, parental satisfaction, and procedure time. A random-effects network meta-analysis estimated relative effects with 95% confidence intervals (CI), using Gomco™ or Mogen™ as reference devices. Analyses were performed in R.

**Results:**

Ten trials including 3,984 infants from Africa, the Middle East, and the United States were analyzed. No device differed significantly from Gomco™ in total adverse events: Plastibell™ (risk ratios, [RR] 1.53, 95% CI 0.56–4.21), ShangRing™ (RR 2.70, 95% CI 0.31–23.66), Mogen™ (RR 3.83, 95% CI 0.97–15.17), and AccuCirc™ (RR 9.62, 95% CI 0.24–388.45). Plastibell™ was associated with a higher infection risk versus Gomco™ (RR 4.25, 95% CI 1.43–12.65). No significant differences were observed in bleeding, redundant skin, adhesions, or parental satisfaction. Mogen™ and ShangRing™ had shorter procedure times than Gomco™ (mean difference [MD] −3.29 minutes, 95% CI −4.82 to −1.76; MD −3.39 minutes, 95% CI −6.11 to −0.67).

**Conclusions:**

Neonatal circumcision devices demonstrate generally comparable safety profiles, though Plastibell™ carries elevated infection risk. Mogen™ and ShangRing™ offer procedural efficiency advantages. Device selection should prioritize provider expertise, infection control capabilities, and programmatic efficiency requirements alongside safety considerations.

## INTRODUCTION

Neonatal and early infant male circumcision is among the most frequently performed surgical procedures worldwide, with an estimated 30% of males circumcised globally (1). In many regions, including sub-Saharan Africa, the Middle East, and North America, circumcision is performed during the neonatal period or early infancy for cultural, religious, or medical reasons (2). Clinically, early circumcision has been associated with reduced risks of urinary tract infections during infancy and long-term protection against sexually transmitted infections, including human immunodeficiency virus (HIV) (3, 4).

Several devices are routinely used for neonatal circumcision, including the Gomco™ clamp, Plastibell™, Mogen™ clamp, ShangRing™, and AccuCirc™. These devices vary substantially in design and technique. For example, the Gomco™ and Mogen™ clamps require immediate foreskin excision, whereas Plastibell™ and ShangRing™ involve delayed tissue necrosis (5). Reported complication rates across randomized controlled trials (RCTs) range from less than 1% to more than 10%, depending on device type, operator experience, and outcome definitions (5-7). Procedure time also varies considerably, with mean durations reported as short as 3–5 minutes for clamp-based devices and longer for ring-based techniques (8).

Although numerous RCTs have compared individual circumcision devices, most studies are limited by small sample sizes and restricted head-to-head comparisons. The largest multicenter trial to date enrolled more than 1,300 infants and compared ShangRing™ with Mogen™, demonstrating comparable safety but differences in procedural characteristics and acceptability, including variations in operative time, need for device removal, post-procedural follow-up requirements, and caregiver and provider satisfaction (9).

Other trials have reported conflicting findings regarding bleeding, infection, and cosmetic outcomes, making it difficult to draw definitive conclusions (6, 7). Moreover, prior reviews have often pooled neonates with older children or adults, despite important anatomical and physiological differences that may influence outcomes (10, 11).

Given the limited number of large head-to-head trials and the fragmented nature of the available evidence, a comprehensive comparative evaluation of circumcision devices remains lacking. Network meta-analysis (NMA) enables simultaneous comparison of multiple interventions by integrating direct and indirect evidence, allowing estimation of relative effects and ranking of competing devices even in the absence of head-to-head trials (12). Accordingly, we conducted a systematic review and NMA of randomized controlled trials comparing commonly used circumcision devices in neonates and infants within the first three months of life, a restriction that improves clinical homogeneity and relevance to early circumcision practice.

## MATERIALS AND METHODS

### Study design

This study was conducted as a systematic review and network meta-analysis comparing commonly used neonatal circumcision devices. The review was performed and reported in accordance with the Preferred Reporting Items for Systematic Reviews and Meta-Analyses (PRISMA) and Cochrane handbook guidelines (13, 14). The protocol was registered in PROSPERO (CRD420261305036).

### Literature search strategy

A comprehensive literature search was conducted in PubMed, EMBASE, Web of Science, and SCOPUS from database inception to January 2026. Terms included "circumcision" and device-related key words such as "circumcision device," "circular circumcision device," "disposable circumcision device," "in situ circumcision," and specific device names including Plastibell™, Shang Ring, PrePex, Tara KLamp, Ali's clamp, circular stapler, circumcision stapler, and compression device. Database-specific adaptations were applied for each platform. Reference lists of relevant articles were manually screened to identify additional eligible studies.

### Eligibility criteria

Studies were included if they met the following criteria: (1) RCTs; (2) enrolled neonates or infants within the first three months of life undergoing circumcision; (3) compared at least two circumcision devices of interest; and (4) reported one or more outcomes related to safety, complications, parental satisfaction, or procedural characteristics; and (5) were published in English. Studies were excluded if they involved adult populations, enrolled infants older than three months, used non-randomized designs, included control groups without device comparison, were published in languages other than English, or were conference abstracts without full-text data.

### Study selection

After removal of duplicate records, titles and abstracts were independently screened by two reviewers. Full texts of potentially eligible studies were assessed for inclusion. Disagreements were resolved through discussion or consultation with a third reviewer. Reasons for exclusion at the full-text stage were documented.

### Data extraction

Data were independently extracted by two reviewers using a standardized data collection form. Extracted information included study characteristics, participant characteristics (age, weight, gestational age, HIV exposure), device types, follow-up duration, and reported outcomes. When required, corresponding authors were contacted for missing or unclear data.

### Risk of bias assessment

Risk of bias was assessed independently by two reviewers using the Cochrane Risk of Bias 2.0 tool (15). Bias was evaluated across five domains: randomization process, deviations from intended interventions, missing outcome data, outcome measurement, and selective reporting. Each study was classified as having low risk, some concerns, or high risk of bias. Discrepancies were resolved by consensus.

### Data synthesis and statistical analysis

A random-effects network meta-analysis was performed to compare all devices simultaneously while preserving within-study randomization. Gomco™ or Mogen™ were used as the reference comparator depending on outcome availability. Effect estimates were presented as pooled risk ratios (RRs) or mean differences (MDs) with corresponding 95% confidence intervals (CIs). Ranking probabilities were summarized using P-scores to estimate the relative performance of each device. All statistical analyses were conducted using R programming software. Statistical heterogeneity was assessed using the I^2^ statistic and Cochran's Q test within designs. Inconsistency between direct and indirect evidence was evaluated using design-by-treatment interaction models when applicable.

## RESULTS

**Figure 1:**
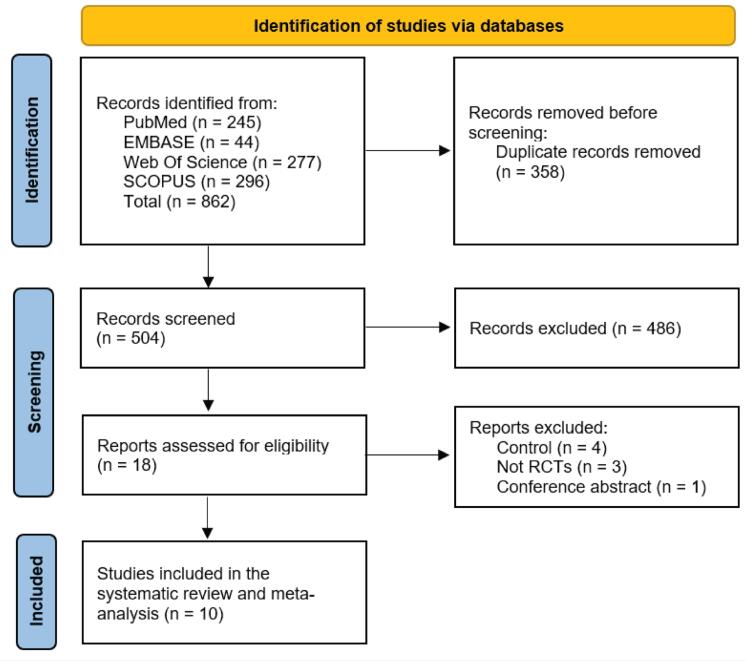
PRISMA Flow Diagram of Study Selection.

A total of 862 records were identified from PubMed, EMBASE, Web of Science, and SCOPUS. After removing 358 duplicate records, 504 studies were screened, of which 486 were excluded. Eighteen reports were assessed for eligibility. Eight studies were excluded for predefined reasons: use of control groups (n = 4), not randomized controlled trials (n = 3), and conference abstracts (n = 1). Ultimately, 10 studies were included in the meta-analysis (6-9, 16-21) (Figure-1).

### Baseline and summary of the included studies

The 10 included RCTs were conducted between 2002 and 2024 across Africa, the Middle East, and the United States. Sample sizes ranged from 59 infants to 1,378 participants, with the largest trial comparing ShangRing™ and Mogen™ devices (689 infants per group) (9). All procedures were performed in the neonatal period or early infancy, with mean ages generally between 3 and 28 days. Mean infant weights were consistently around 3.2–4.2 kg. Gestational age, when reported, indicated predominantly term infants (mean ∼39–40 weeks). Several African studies included HIV-exposed infants, ranging from 16% to 41%. Devices evaluated included Gomco™, Plastibell™, Mogen™, ShangRing™, and AccuCirc™. Follow-up durations varied from immediate assessment to 6 months. Outcomes commonly assessed safety, complications, pain, healing, cosmesis, and caregiver satisfaction, with comparable baseline characteristics across intervention groups (Table-1).

**Table 1:** Baseline and Summary of the included studies.

ID	Design	Country	Registration	Follow-up	Primary outcomes	Device	Sample size (n)	Age at circumcision, days, Mean ± SD	Weight at circumcision, Kg, Mean ± SD	Infant exposure to HIV, %	gestational age in weeks Mean ± SD
**Alsowayan et al. 2024**	RCT	Saudi Arabia	IRB-PCS201901-042	1–3 weeks	Parent/physician satisfaction	Gomco	98	<3 months	Mean 4.19	NR	NR
						Plastibell	92				
**Ibiyeye et al. 2024**	RCT	Nigeria	NR	7 d & 4 weeks	Healing, cosmesis	Gomco	50	14.08 ± 7.19	3.71 ± 0.65	NR	39.54 ± 1.17
						Plastibell	50	15.17 ± 6.16	3.71 ± 0.65		38.58 ± 1.22
**Basourakos et al. 2022**	Non-inferiority RCT	East Africa	NCT03338699	Until healing	Safety	ShangRing	689	26 ± 23.8	4.1 ± 0.89	NR	NR
						Mogen	689	26.3 ± 22.3	4.2 ± 1.04		
**Bawazir et al. 2019**	RCT	Saudi Arabia	NR	1 week & 6 months	Complications	Gomco	410	Mean 19 days	NR	NR	NR
						Plastibell	383				
**El-Asmar et al. 2017**	RCT	Egypt	ACTRN12616000190404	1 & 4 weeks	Pain, complications	Gomco	50	NR	NR	NR	NR
						Plastibell	50				
**Mavhu et al. 2015**	Non-inferiority RCT	Zimbabwe	PACTR201301000465398	14 days	Safety, acceptability	AccuCirc	100	8 ± 8	3.2 ± 0.38	18 (18%)	40 ± 0.83
						Mogen	50	8 ± 11.5	3.2 ± 0.48	8 (16%)	40 ± 1
**Sinkey et al. 2015**	RCT	USA	NCT01726036	Short-term	Neonatal pain	Gomco	137	NR	3.45 ± 5.15	NR	39.44 ± 1.08
						Mogen	137		3.4 ± 4.18		39.29 ± 1.14
**Bowa et al. 2013**	RCT	Zambia	NR	1 & 6 weeks	Safety, adverse events	Mogen	216	27.3 ± 5.97	3.57 ± 0.6	45 (20.8%)	NR
						Gomco	206	28.3 ± 6.7	3.6 ± 0.6	45 (20.8%)	
						Plastibell	218	28.7 ± 7.5	3.6 ± 0.6	59 (27.1%)	
**Plank et al. 2013**	RCT	Botswana	NCT00971958	6 weeks, 4 months	Adverse events	Mogen	153	5.7 ± 6.74	3.2 ± 0.45	62 (41.1%)	39.3 ± 2.45
						Plastibell	147	3 ± 2.45	3.3 ± 0.45	51 (35.4%)	38.7 ± 0.75
**Taeusch et al. 2002**	RCT	USA	NR	Immediate	Pain & duration	Mogen	30	NR	NR	NR	NR
						Plastibell	29				

RCT, randomized controlled trial; NR, not reported; d, days, SD, standard deviation; kg, kilograms; HIV, human immunodeficiency virus.

### Quality assessment

**Figure 2:**
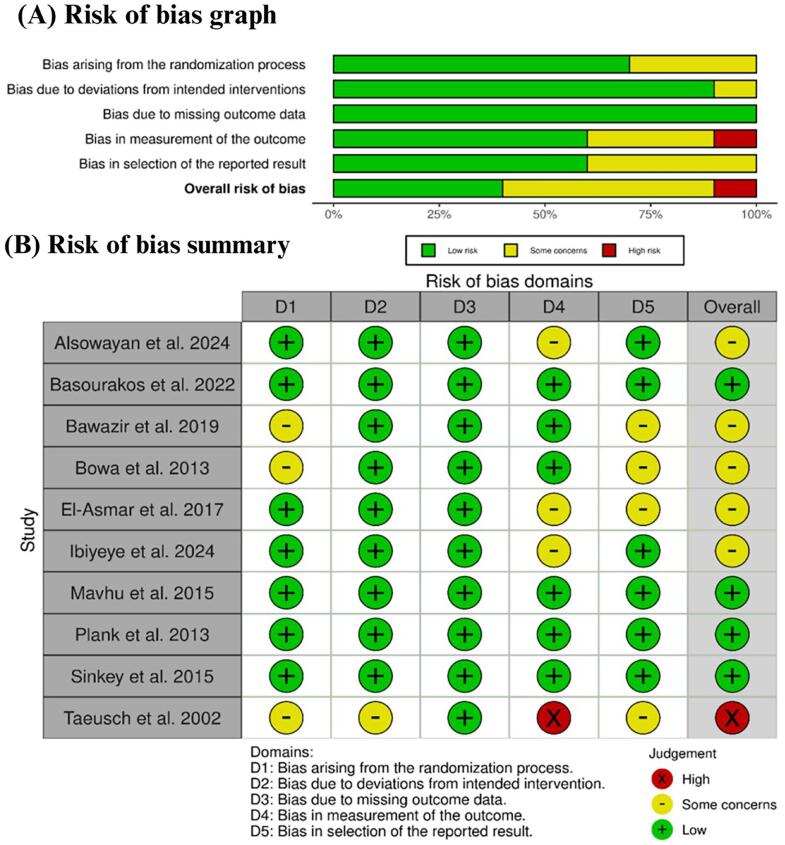
(A) Risk of bias graph and (B) Risk of bias summary.

The risk of bias assessment across the 10 included studies showed generally good methodological quality. Four studies demonstrated an overall low risk of bias, reflecting good randomization, outcome measurement, and reporting (9, 19-21). Some concerns were identified in five studies, particularly in the randomization process and outcome measurement (6, 7, 16-18). One early study was judged to have a high overall risk of bias, mainly due to limitations in outcome measurement and reporting (8). Overall, the evidence base was considered moderate to high quality (Figures 2A and 2B).

### Outcomes

#### Total adverse events

**Figure 3:**
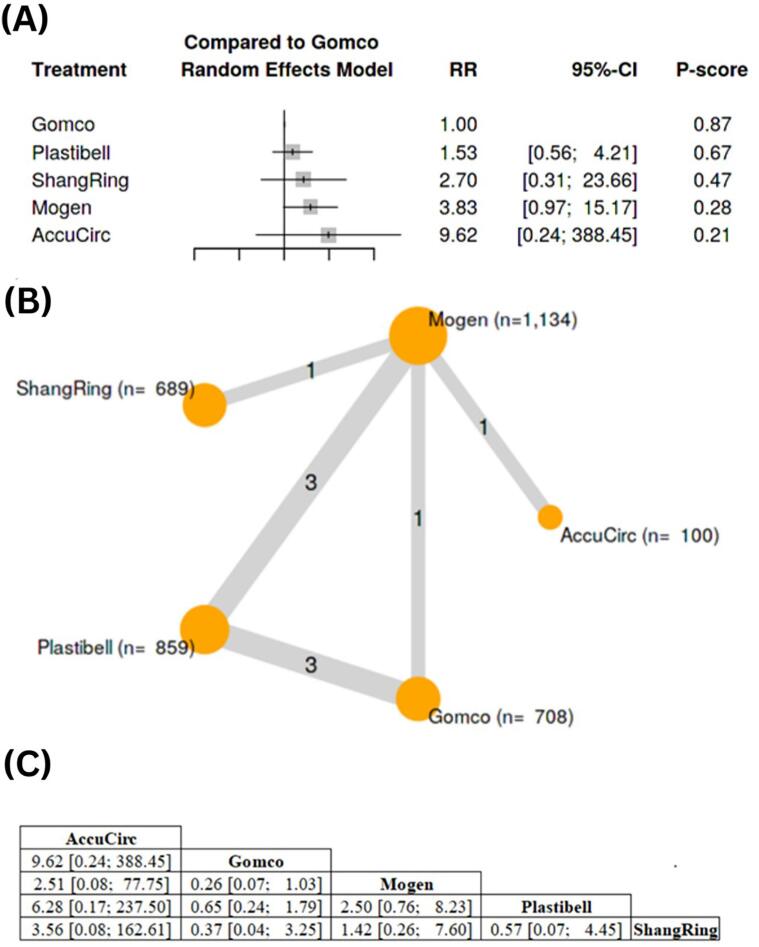
Total Adverse Events, (A) Network Meta-analysis, (B) Network graph, and (C) Net league

Using Gomco™ as the reference, no statistically significant differences were observed in total adverse events across devices, as follows: Plastibell™ (RR 1.53, 95% CI 0.56–4.21), ShangRing™ (RR 2.70, 95% CI 0.31–23.66), Mogen™ (RR 3.83, 95% CI 0.97–15.17), and AccuCirc™ (RR 9.62, 95% CI 0.24–388.45). Head-to-head comparisons among AccuCirc™, Mogen™, Plastibell™, and ShangRing™ similarly showed no significant differences. Substantial heterogeneity was present (I^2^ = 78.5%), mainly due to inconsistency between direct and indirect evidence (Q = 18.6, p < 0.001) (Figures 3A, 3B and 3C).

#### Bleeding risk

**Figure 4:**
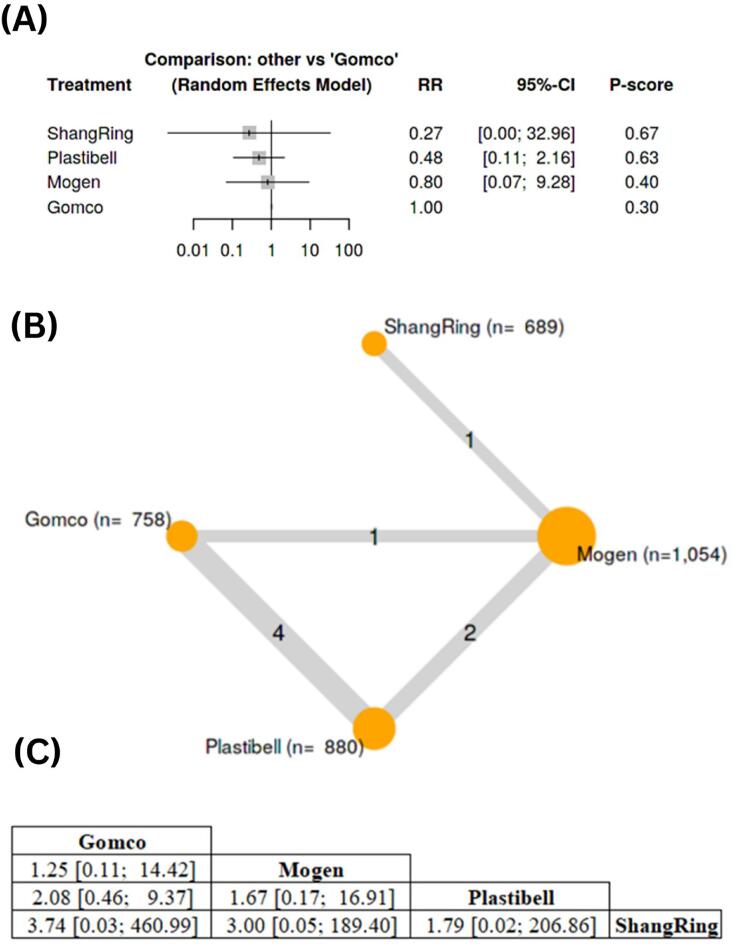
Bleeding Risk, (A) Network Meta-analysis, (B) Network graph, and (C) Net league

Similarly, compared with Gomco™, no significant differences were found in the risk of bleeding across devices. ShangRing™ showed a risk ratio of 0.27 (95% CI 0.00–32.96), Plastibell™ a risk ratio of 0.48 (95% CI 0.11–2.16), and Mogen™ a risk ratio of 0.80 (95% CI 0.07–9.28). Head-to-head comparisons among ShangRing™, Plastibell™, and Mogen™ similarly demonstrated no significant differences in bleeding risk. High heterogeneity was present (I^2^ = 77.6%) (Figures 4A, 4B, and 4C).

#### Infection risk

**Figure 5:**
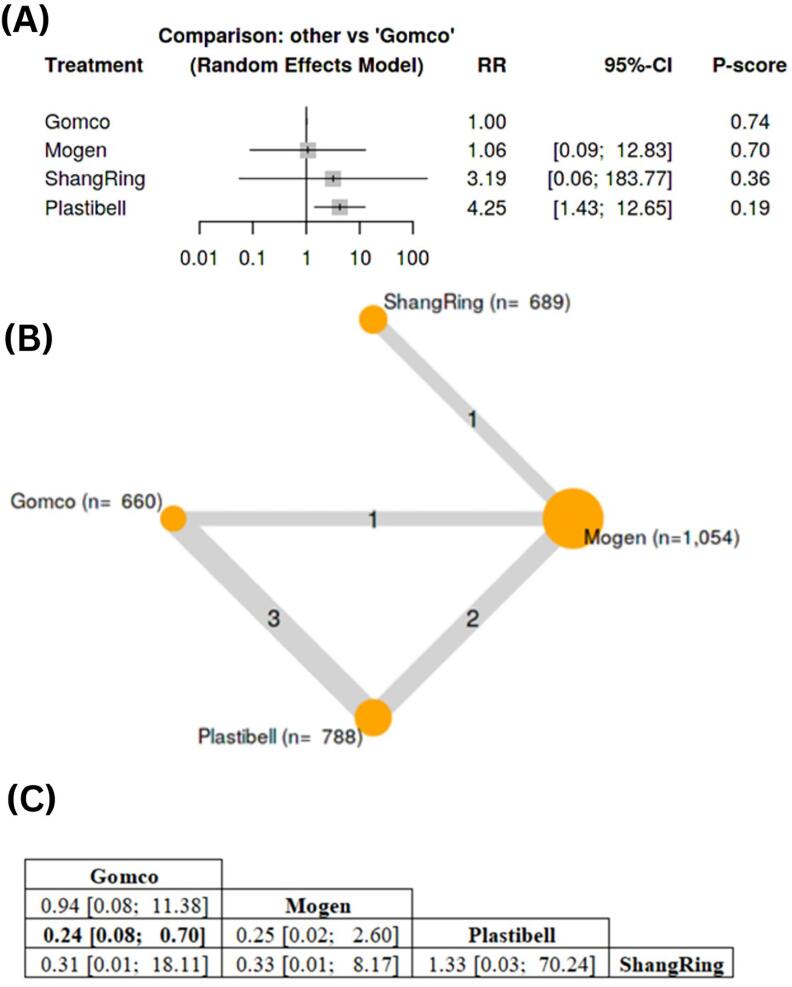
Infection Risk, (A) Network Meta-analysis, (B) Network graph, and (C) Net league

Compared with Gomco™, no statistically significant difference in infection risk was observed for Mogen™ (RR 1.06, 95% CI 0.09–12.83) or ShangRing™ (RR 3.19, 95% CI 0.06–183.77), whereas Plastibell™ was associated with a significantly higher risk of infection (RR 4.25, 95% CI 1.43–12.65). No significant differences were observed in head-to-head comparisons. Heterogeneity was negligible, with no evidence of within-design variability or inconsistency between direct and indirect results (I^2^ = 0%) (Figures 5A, 5B and 5C).

**Figure 6:**
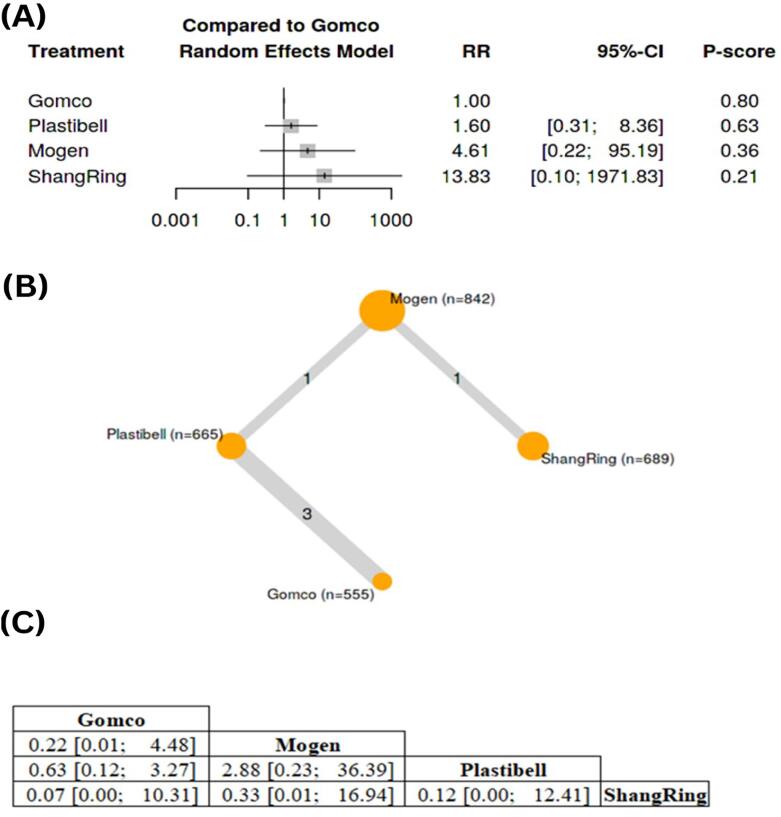
Residual (Redundant) Skin, (A) Network Meta-analysis, (B) Network graph, and (C) Net league

No statistically significant differences were observed between devices in terms of redundant skin. No major heterogeneity was detected within study designs (Q = 5.5, p = 0.064), while overall heterogeneity was moderate (I^2^ = 63.6%) (Figures 6A, 6B and 6C).

#### Adhesions

**Figure 7:**
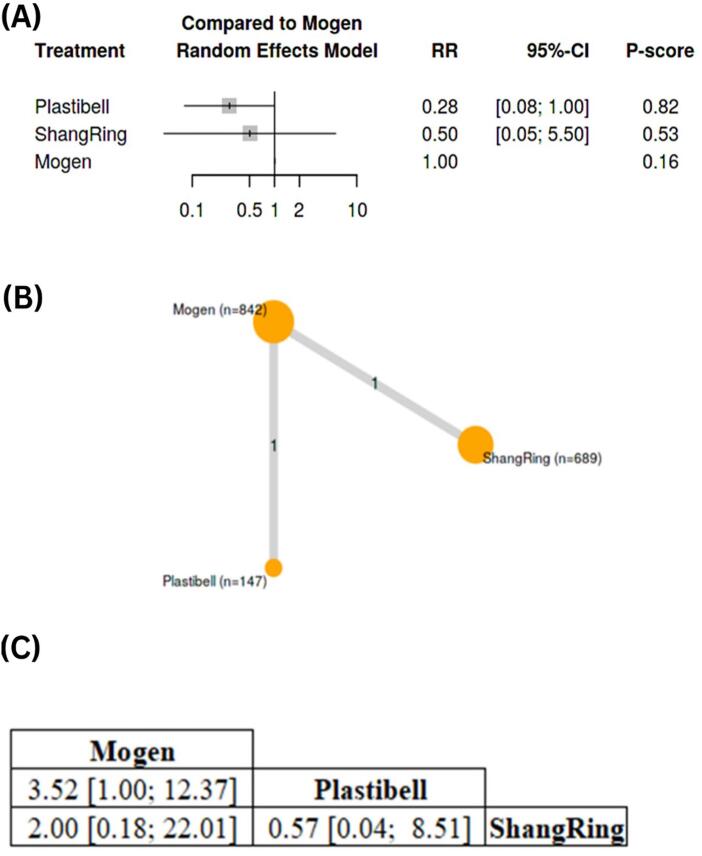
Adhesions, (A) Network Meta-analysis, (B) Network graph, and (C) Net league

For adhesions, Mogen™ was used as the reference (RR = 1.00; P-score = 0.16). Lower risks were estimated for Plastibell™ (RR 0.28, 95% CI 0.08–1.00) and ShangRing™ (RR 0.50, 95% CI 0.05–5.50), with no statistically significant differences observed. Heterogeneity could not be formally assessed because only two studies were included in the analysis (Figures 7A, 7B, and 7C).

#### Parental satisfaction rate

**Figure 8:**
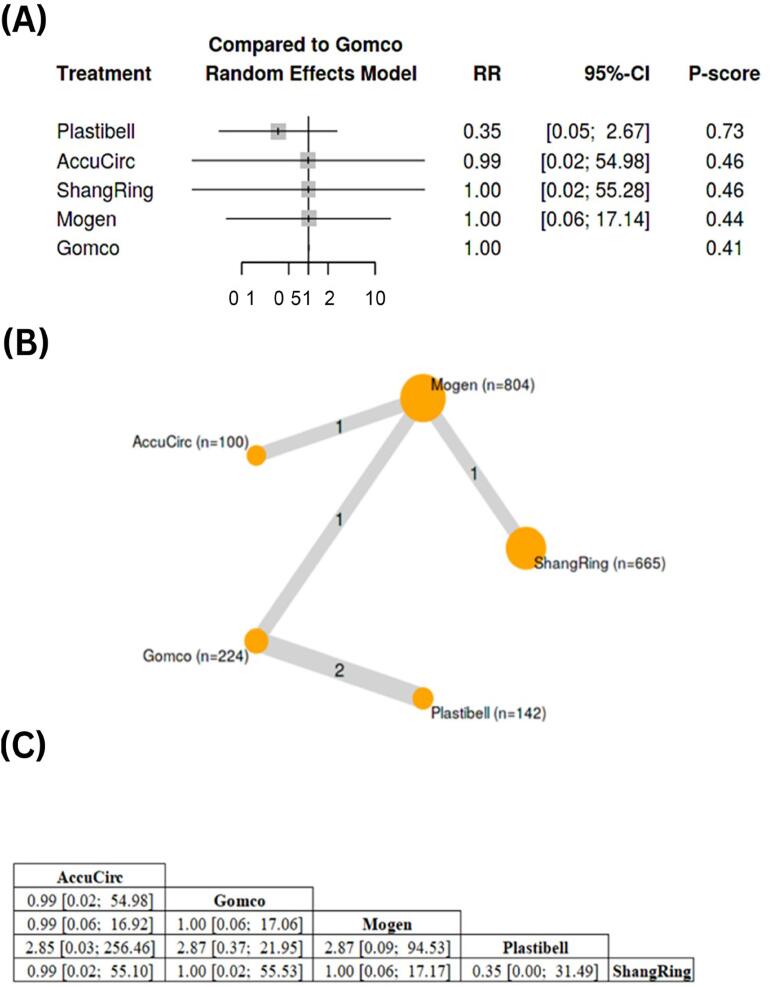
Parental Satisfaction Rate, (A) Network Meta-analysis, (B) Network graph, and (C) Net league

Compared with Gomco™, estimated parental satisfaction was comparable for Plastibell™ (RR 0.35, 95% CI 0.05–2.67; P-score 0.73), AccuCirc™ (RR 0.99, 95% CI 0.02–54.98), ShangRing™ (RR 1.00, 95% CI 0.02–55.28), and Mogen™ (RR 1.00, 95% CI 0.06–17.14). However, substantial heterogeneity was observed (I^2^ = 97.2%), because of significant within-design variability (Figures 8A, 8B and 8C).

#### Procedure Time (minutes)

**Figure 9:**
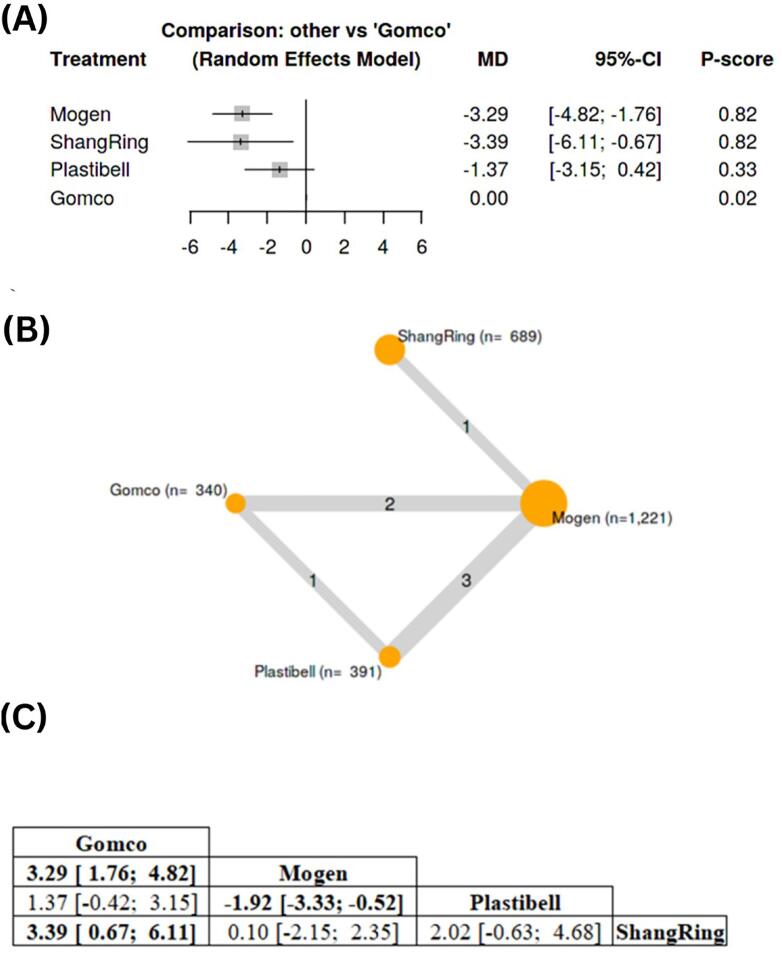
Procedure Time, (A) Network Meta-analysis, (B) Network graph, and (C) Net league

Compared to Gomco™, both Mogen™ (MD −3.29 minutes, 95% CI −4.82 to −1.76; P-score 0.82) and ShangRing™ (MD −3.39 minutes, 95% CI −6.11 to −0.67; P-score 0.82) were associated with shorter procedure times. Plastibell™ did not show time reduction (MD −1.37 minutes, 95% CI −3.15 to 0.42). Direct comparison between Mogen™ and Plastibell™ favored Mogen™, indicating a shorter procedure time (MD −1.92, 95% CI −3.33 to −0.52). Heterogeneity was observed within study designs (Q = 10.6, p = 0.001), with no inconsistency between direct and indirect evidence (Figures 9A, 9B and 9C).

## DISCUSSION

This network meta-analysis synthesized evidence from 10 RCTs involving neonates and infants within the first three months of life to compare commonly used circumcision devices. Our findings refine current assumptions about device safety and performance.

The most important finding was the absence of significant differences in total adverse events across most devices. This overall safety equivalence is reassuring for clinicians and parents facing device selection decisions. However, further analysis of specific complications revealed important distinctions. Plastibell™ demonstrated a notable elevated infection risk compared to Gomco™, a finding that warrants clinical attention despite the device's widespread use. The mechanism behind this increased infection risk likely relates to the Plastibell™'s design principle of delayed tissue necrosis, where the plastic ring remains in place for several days post-procedure (22). This extended period, during which the Plastibell™ ring typically remains in place for 5–10 days (mean ≈8.7 days in infants under three months) (23), creates a potential site for bacterial colonization beneath the device, particularly if hygiene protocols are not maintained or if the ring does not detach as expected.

In contrast, bleeding complications showed no significant differences across devices, suggesting that when properly applied by trained providers, all devices provide adequate hemostasis. The lack of differences in redundant skin and adhesions further supports that device choice may be less critical than operator skill and experience for these specific outcomes (24). Perhaps most relevant for clinical workflow, both Mogen™ and ShangRing™ demonstrated significantly shorter procedure times compared to Gomco™, approximately 3 minutes faster on average. For busy clinical practices or resource-limited settings conducting high-volume circumcision programs, this efficiency gain could translate into substantial time savings. The mechanism is straightforward: both Mogen™ and ShangRing™ require fewer procedural steps and simpler application techniques than the more complex Gomco™ clamp assembly (21).

Our findings both align with and extend previous systematic syntheses while highlighting important distinctions. Meta-analyses comparing device-assisted with conventional circumcision techniques have consistently reported shorter operative times and reduced bleeding with device-based approaches. For example, Kafagi et al. (2025) demonstrated improved procedural efficiency with device-assisted techniques, findings that are consistent with our results on operative duration (11). However, their inclusion of older children limits direct comparability with our exclusively neonatal population.

Similarly, Güler et al. (2022) reported that ring devices were associated with shorter surgical duration and higher satisfaction compared with classic open methods (25). Notably, they identified an increased hemorrhage risk with Plastibell™ relative to open procedures. In our analysis, Plastibell™ also demonstrated an elevated complication profile, although this manifested primarily as increased infection risk rather than bleeding, suggesting device-specific patterns of adverse events in early infancy.

Broader evaluations of circumcision safety further contextualize our findings. Shabanzadeh et al. (2021) reported an overall complication rate of 3.84% across all ages, with higher risks observed in therapeutic procedures (26). Consistent with established evidence, Weiss et al. (2010) demonstrated substantially lower complication rates in neonates compared with older children (median 1.5% vs 6%) (27). By restricting our analysis to neonatal nontherapeutic circumcision, we observed generally lower complication rates, reinforcing the safety advantage of early intervention.

Importantly, while prior meta-analyses such as Huo et al. (2017) concluded that disposable circumcision devices and conventional methods have comparable overall safety profiles (10), these studies relied primarily on pairwise comparisons. In contrast, our network meta-analysis provides device-specific relative effect estimates and hierarchical rankings, allowing simultaneous comparison of multiple commonly used devices and offering more granular evidence to inform clinical decision-making.

Our findings have several important implications for clinical practice and public health programming. First, the overall safety equivalence across most devices empowers clinicians to select instruments based on their training, comfort level, and procedural context rather than safety concerns alone. An exception is the higher infection risk associated with the Plastibell™ device, which warrants careful consideration, particularly in settings with limited follow-up capacity or where caregiver education on device hygiene may be inadequate.

Second, shorter procedure times with Mogen™ and ShangRing™ devices hold particular relevance for resource-constrained settings implementing large-scale early infant male circumcision programs for HIV prevention in sub-Saharan Africa. Third, time savings of 3-4 minutes per procedure could enable substantially increased daily case volumes without compromising quality. However, in circumcision programs for HIV prevention our parental satisfaction data showed no significant differences between devices, possibly reflecting the multifactorial nature of satisfaction.

Finally, the high heterogeneity observed in several outcomes suggests that some differences between devices may be influenced by variability in study design, outcome definitions, provider expertise, and follow-up duration rather than true device-related effects. This variability, along with wide confidence intervals, reduces the certainty of pooled estimates and limits the reliability and consistency of effect direction. Therefore, findings should be interpreted with caution, as standardization of surgical technique, provider training, and postoperative care may be as important as device selection in optimizing outcomes.

This network meta-analysis represents the most comprehensive simultaneous comparison of neonatal circumcision devices to date, employing a rigorous methodology including systematic literature search, independent dual review, and validated risk-of-bias assessment. By restricting our analysis to the first three months of life, we achieved greater clinical homogeneity than previous reviews that pooled neonates with older children or adults. The network approach enabled both direct and indirect comparisons, generating relative effect estimates even for device pairs rarely compared head-to-head in primary trials.

However, several limitations warrant acknowledgment. First, substantial statistical heterogeneity in some outcomes suggests unmeasured variability across studies in patient populations, surgical techniques, or outcome definitions. Second, the small number of trials for certain device comparisons (particularly AccuCirc™) limited precision for some estimates, reflected in wide confidence intervals. Third, follow-up duration varied considerably across studies, potentially affecting late complication detection. Finally, all included studies were conducted in hospital or clinic settings; generalizability to traditional or community-based circumcision programs remains uncertain.

This network meta-analysis demonstrates that commonly used circumcision devices for neonates and early infants offer generally comparable safety profiles, with the notable exception of increased infection risk with Plastibell™. Mogen™ and ShangRing™ provide procedural efficiency advantages through significantly shorter operating times, making them particularly attractive for high-volume settings. Device selection should be guided by provider expertise, procedural context, infection control capabilities, and efficiency requirements rather than fears of safety concerns.

We recommend that circumcision programs emphasize comprehensive provider training, standardized technique, and strong infection prevention protocols regardless of device choice. Future research should prioritize large-scale pragmatic trials with extended follow-up to capture late complications, economic analyses comparing device costs and efficiency, and implementation studies examining optimal training and quality assurance strategies. Particular attention should be directed toward understanding and mitigating the elevated infection risk associated with Plastibell™, potentially through enhanced caregiver education and closer follow-up during the critical period before ring detachment.

## Data Availability

All data generated or analysed during this study are included in this published article
